# *Meloidogyne Incognita* Management using Fumigant and Non-fumigant Nematicides on Sweet Potato

**DOI:** 10.2478/jofnem-2022-0026

**Published:** 2022-07-29

**Authors:** Chang Liu, Zane Grabau

**Affiliations:** 1 Entomology and Nematology Department, University of Florida, Gainesville, FL 32611 Florida

**Keywords:** 1,3-dichloropropene, fluazaindolizine, fluopyram, free-living nematode, fumigant, *Ipomoea batatas*, management, *Meloidogyne incognita*, nematicide, oxamyl, southern root-knot nematode, sweet potato

## Abstract

Southern root-knot nematode (SRKN, *Meloidogyne* incognita) is a major pest of sweet potato, and nematicides are needed to manage this nematode. The objectives of this study were to assess the efficacy of fluazaindolizine, a new non-fumigant nematicide, in comparison with the fumigant nematicide 1,3-dichloropropene (1,3-D) and non-fumigant nematicides fluopyram and oxamyl for (1) SRKN management, (2) impacts on free-living nematodes, and (3) sweet potato yield in field trials. Among all nematicides, 1,3-D at 84 kg/ha most consistently (2 of 3 years) managed SRKN soil populations and improved yield. Fluazaindolizine at 2.24 kg/ha and fluazindolizine at 1.12 kg/ha plus oxamyl at 2.14 kg/ha managed SRKN populations and improved yield in 1 of 3 years, whereas fluazaindolizine alone at 1.12 kg/ha only decreased SRKN populations. Fluopyram at 238 g/ha did not affect SRKN or yield. Nematicide application also had non-target effects on free-living nematodes with 1,3-D reducing abundances relative to untreated most frequently (2018 and 2020), but other nematicides also reducing free-living nematode abundances in 2020. In summary, 1,3-D is the most consistent option for SRKN control on sweet potato, but fluazaindolizine, oxamyl or combinations of the two products can also be effective.

Sweet potato (*Ipomoea batatas*) is an important food source with its large, sweet, and starchy root. The production of sweet potato in the United States has increased from 38,364 ha in 2000 to 63,454 ha in 2020, and the production value has increased from 210 million to 726 million dollars (USDA NASS, 2021). Sweet potatoes are frequently damaged by southern root-knot nematode (SRKN, *Meloidogyne incognita*) and this pest is commonly found in the major regions of sweet potato production (Wendimu, 2021). It can infect sweet potato tubers, causing galling on the tuber surface, and prevent the sweet potato tuber from being marketable (Clark *et al*., 2013; Quesada-Ocampo, 2018; Wendimu, 2021).

Currently, there are few SRKN management methods available for sweet potato growers, with options including crop rotation, resistant cultivars, and nematicide application. Among nematicides, there are both fumigants and non-fumigants registered for nematode management on sweet potato. Since nematodes are soil-borne pathogens, nematode management before planting is crucial. Fumigants applied before planting have proven to be effective in the control of a wide spectrum of nematodes. In the United States, commonly used fumigants registered use on sweet potato include 1.3-dichloropropene (1,3-D), metam sodium, and metam potassium. With the growing concerns for environmental safety, in the past decades, several major nematicides (e.g., methyl bromide) have been phased out, and increasing regulatory pressure on older chemistries has increased emphasis on discoveries of new non-fumigant nematicides (Desaeger *et al*., 2020). While fumigants move through the soil as gas, non-fumigant nematicides are applied in liquid or solid formulations, and move through the soil as a liquid rather than a gas. There are a few non-fumigant nematicides registered for use on sweet potato in the United States, including the older chemicals ethoprop and oxamyl. However, additional effective non-fumigant nematicides, especially those with fewer non-target effects are always in demand.

In recent years, several new fluorinated non-fumigant nematicides became available, including fluensulfone, fluopyram, and fluazaindolizine, and have been found to be effective in managing root-knot nematodes (RKN) on several vegetable crops in greenhouses and field studies (Becker *et al*., 2019; Desaeger and Watson, 2019; Khanal and Desaeger, 2020; Grabau *et al*., 2021). These new discoveries may provide growers alternatives to fumigants for nematode management. Peiris *et al*. (2021) reported that fluensulfone can effectively decrease RKN density in the soil and increase marketable sweet potato yield. Waisen *et al*. (2021) reported early that fluopyram chemigation can reduce RKN population in sweet potato production. Both fluopyram and fluensulfone are available for use in sweet potato production in the United States, but fluazaindolizine is not yet available.

Fluazaindolizine is a novel sulfonamide nematicide (Lahm *et al*., 2017). The mode of action remains unknown, but lab studies showed that it has adverse effect on RKN motility and infectivity (Thoden and Wiles, 2019; Watson, 2021). Several field studies on various crops including tomato, carrot, and squash have shown the potential of fluazaindolizine as a new nematicide against RKN (Becker *et al*., 2019; Desaeger and Watson, 2019; Talavera *et al*., 2021; Zhang *et al*., 2021). However, little is known about the field performance of fluazaindolizine against SRKN on sweet potato, so it was investigated in this study.

Free-living nematodes are important indicators of soil health since they can contribute to improving soil nutrient cycling (Holajjer *et al*., 2016; Trap *et al*., 2016), pathogen management (Khan and Kim, 2005; Kanfra *et al*., 2018), and ecological flexibility (Jiang *et al*., 2018; Schratzberger *et al*., 2019), which are important components of soil productivity. The free-living nematode profile is also a reflection of soil biodiversity since there are multiple trophic groups involved (Ferris *et al*., 2001). Many nematicides are reported to reduce free-living nematode diversity and abundance (Wang, 2005; Waldo *et al*., 2019; Grabau *et al*., 2020). Because of these factors, investigating the influence of nematicides on free-living nematodes is increasingly an important component of evaluating new nematicides.

The objectives of this study are to investigate the effect of fluazaindolizine at different rates and in combination with older non-fumigant nematicides on management of SRKN on sweet potato and non-target free-living nematodes, relative to other fumigant and non-fumigant nematicides.

## Materials and Methods

### Field site and trial maintenance

To assess these objectives, three field trials were carried out at the North Florida Research and Education Center-Suwannee Valley in Live Oak, Florida (30°18¢11.6^2^N 82°53¢48.6^2^W). Trials were conducted in 2018, 2019, and 2020 in three different fields at the center. Soil at the sites was Chipley sand (91% sand, 6.8% silt, 2.4% clay, and 3.1% organic matter). In all experiments, fertilization, irrigation (supplied by overhead lateral line or center pivot irrigation), and herbicide applications were uniform across the trial and based on University of Florida recommendations (Mylavarapu *et al*., 2017; Beuzelin *et al*., 2021). No fungicides or insecticides were applied in any year. Trial sites at the center were selected for presence of SRKN. The trial sites in 2018 and 2020 were grown to sorghum (*Sorghum bicolor*) the previous year and peanut (*Arachis hypogea*), field corn (*Zea mays*), and winter rye (*Secale cereale*) cover crop were among previous crops. The 2019 site was grown to cotton (*Gossypium hirsutum*) the previous year and had a history of both vegetable and agronomic crops.

### Experiment design and treatment applications

This study was a randomized complete block design with five replicates and nematicides as the single factor (Table 1). Each field plot consisted of two beds of sweet potatoes with 1 m space from row center to center and 0.6 m bed tops. Within beds, plant spacing was 30 cm. Plots were 9.1 m long. In 2018, 2019, and 2020, 1,3-D was applied 3–4 wk before planting (Table 2) via a fumigation rig with shanks spaced 30 cm apart. The rig was configured with five coulters to open traces immediately in front of the five shanks with press wheels behind each shank to seal traces, and 1,3-D was released at 25 cm deep in the soil profile. The area fumigated was 1.83 m wide, which covered the area where soil was pulled into beds. Non-fumigant nematicides were applied as a broadcast application 8–11 d before planting via CO_2_ powered backpack sprayer except that fluopyram was applied via drench at planting in 2020 (Table 1). For broadcast sprays, non-fumigant nematicides were applied using a wand with three nozzles spaced 0.6 m apart to produce a 2-m spray band at a solution application rate of 150 L/ ha. Following non-fumigant nematicide broadcast spray applications, all plots were rototilled at a 15-cm depth to incorporate nematicides then irrigated for further incorporation. In 2020, slips were transplanted, then later in the day, fluopyram was applied as a drench. For 2020 drench application, fluopyram was mixed in water and applied as a broadcast treatment manually with watering cans to selected plots to mimic application via overhead irrigation. The drench volume was 36.1 L/plot or 19,482 L/ha, which is equivalent to 1.96 cm of rain or irrigation. Before planting, 0.6 m wide hills were formed by a hill-forming mechanical discer, and sweet potato slips were planted in bare ground hills. In 2018, a sweet potato cultivar moderately resistant to SRKN (“Covington”) was used, whereas in the SRKN-susceptible cultivars “Beauregard” and “Orleans” were used in 2019 and 2020, respectively. A moderately resistant cultivar was used in 2018 since it is the primary cultivar growers use in this region, but the switch to susceptible cultivars was made in 2019 to evaluate the nematicides under higher SRKN pressure that accompanies the use of a susceptible cultivar.

**Table 1:** Nematicide application treatment rates and application methods in 2018–2020 trials.

Treatment	Product	Active ingredient	Product application rate	Application rate (a.i.)	Application method
1	Untreated control				
2	Salibro^a^	Fluazaindolizine	2.24 L/ha	1.12 kg/ha	Broadcast spray
3	Salibro	Fluazaindolizine	4.48 L/ha	2.24 kg/ha	Broadcast spray
4	Salibro + Vydate L^b^	Fluazaindolizine + oxamyl	2.24 L/ha + 9.35 L/ ha	1.12 kg/ha + 2.14 kg/ha	Broadcast spray
5	Telone II^b^	1,3-D	74 L/ha	84 kg/ha	Broadcast shank fumigation
6	Velum Prime^c^	Fluopyram	499 mL/ha	238 g/ha	Broadcast spray (2018–2019) Drench (2020)
7	Vydate L	Oxamyl	9.35 L/ha	2.14 kg/ha	Broadcast spray

a Salibro was from Corteva Agrisciences (Indianapolis, IN). ^b^Vydate L and Telone II were from Dow Agrisciences (Indianapolis, IN). ^c^Velum Prime was from Bayer CropScience LP (St. Louis, MO).

**Table 2:** Schedule for data collection and trial maintenance in 2018–2020.

Task	2018	2019	2020
Preplant soil sample	May 16 (37 DBP)^a^	May 4 (40 DBP)	May 18 (24 DBP)
Fumigation	May 22 (31 DBP)	May 13 (31 DBP)	May 22 (20 DBP)
Nematicide broadcast spray applications^b^	June 11 (11 DBP)	June 4 (9 DBP)	June 3 (8 DBP)
Sweet potato planted	June 22	June 13	June 11
Midseason soil sample	August 23 (64 DAP)	August 16 (64 DAP)	August 3 (53 DAP)
Harvest soil sample	October 4 (106 DAP)	October 31 (139 DAP)	November 6 (149 DAP)
Sweet potato harvest	October 23 (127 DAP)	November 1 (140 DAP)	November 11 (154 DAP)

a Numbers in parentheses are DBP or DAP. ^b^In 2020, fluopyram nematicide was applied by drench at planting. DAP, days after planting; DBP, days before planting.

### Sweet potato tuber measurements

Sweet potato vines were mowed approximately 5 mon after planting (Table 2), tubers were inverted mechanically the next day, and tubers were picked by hand from both rows of the entire length of the plot. Total sweet potato tuber yield, in weight, was measured. To estimate marketable yield, a random subsample of sweet potato tubers from each plot was collected by filling a 19-L bucket with tubers, because it was not feasible to grade all tubers in each plot. Each tuber in the subsample was graded manually and sorted into the various marketable or unmarketable categories described below. Total weight for each category was calculated based on total yield and proportion weight of each grade category in the subsample on a per plot basis. In 2018, sweet potato subsamples were sorted into marketable and unmarketable categories and weighed. Any tubers <7.6 cm long, <3.8 cm diam., or with quality defects, described below, were considered unmarketable. In 2019 and 2020, tubers were graded to USDA standards (USDA AMS, 2005) with both USDA #1 and USDA #2 considered marketable, but USDA 1 representing the highest quality grade. Unmarketable categories included size outliers and defects due to quality issues. Requirements for USDA 1 were 4.4–8.9 cm diam., <0.5 kg, 7.6–22.9 cm length, free from damage, firm, fairly smooth, fairly clean, and fairly well-shaped. Requirements for USDA 2 were >3.8 cm diameter., <1 kg, firm and free from damage. Any tuber with damage as defined in USDA standards was considered a defect with the vast majority of defects due to damage from wireworms (Coleoptera: Elateridae), juvenile stages of click beetles. Remaining defects were primarily due to mechanical damage from digging or collecting soil samples, and a few miscellaneous defects such as rot. In 2019 and 2020, percent tuber surface galling (0%–100%) was estimated for each of a subsample of 50 harvested tubers. This was estimated visually by a single researcher throughout each trial to increase rating consistency. In 2018, when the resistant cultivar was grown, there was minimal tuber galling at harvest, so formal assessment was not conducted.

### Soil sampling and soil nematode quantification

Soil samples were taken at preplant, midseason (approximately 2 mon after planting), and harvest each year, with precise sampling dates listed in Table 2. Twelve soil cores were collected in the root zone to 30 cm deep with a probe in each plot and homogenized. Soil samples were stored in plastic bags at 4°C for 48 hr maximum before subsequence processing. A 100 cm^3^ subsample soil was taken from each sample and used for nematode extraction by centrifugal-floatation method (Jenkins, 1964). Nematodes were identified to genera for plant-parasitic nematodes, and the quantity of each plant-parasitic nematode and total free-living nematodes were determined immediately after extraction with an inverted light microscope (Zeiss, Primovert) at 400× magnification.

### Statistical analysis

Since the cultivar used varied by year, the data was analyzed separately by year. Southern root-knot nematode abundance, free-living nematode abundance, sweet potato tuber yield by categories, and root galling were used as response variables. Response variables were not transformed since all data met assumptions of homogeneity of variance using Levene’s test (Levene, 1960) and normality of residuals based on graphing (Cook and Weisburg, 1999). Data were statistically analyzed by ANOVA using RStudio (Version 1.2.5019, Boston, MA) and treatment means were separated using Fischer’s LSD (*P =* 0.05) when treatment effects were statistically significant in ANOVA (*P* < 0.05).

## Results

### Nematicide efficacy at managing SRKN

**Figure 1:**
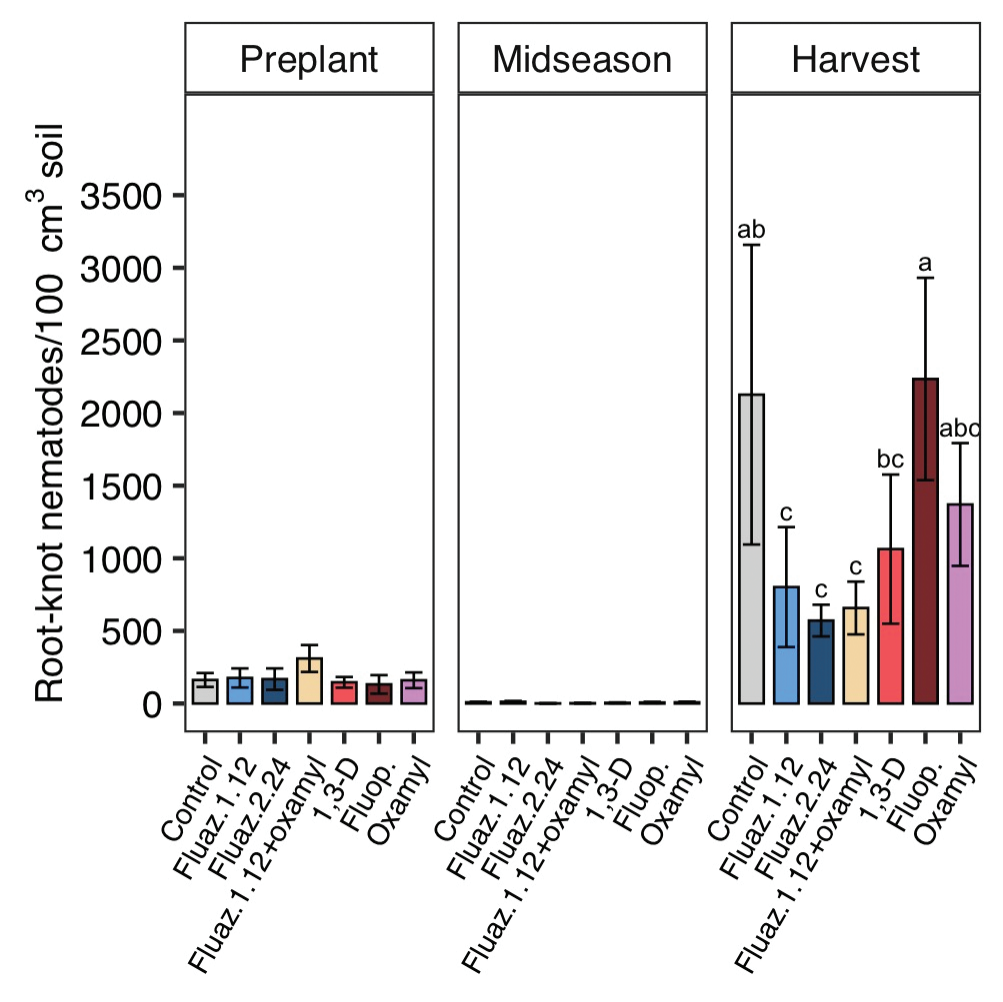
Nematicide effects on *Meloidogyne incognita* J2 soil abundances at preplant, midseason, and harvest in 2018 trials. Within each subfigure, means with different letters are significantly different (*P* < 0.05) based on Fisher’s protected LSD. “Fluaz. 1.12” and “Fluaz. 2.24” indicate fluazaindolizine at 1.12 kg a.i./ha and 2.24 kg a.i./ha, respectively. “Fluop.” Indicates fluopyram.

Southern root-knot nematode was the major plant-parasitic nematode found in soil samples. Ring nematode (*Mesocriconema* sp.) and stubby-root nematode (*Paratrichodorus* sp.) were also found at the experimental sites. Ring and stubby-root nematode soil abundances found at the site were not formally analyzed since their presence was inconsistent and neither nematode is reported to be damaging on sweet potato. In 2018, nematode abundance at the site was 180 SRKN J2/100 cm^3^ soil before planting. Southern root-knot nematode abundance was not affected by treatments at midseason but was affected at harvest in 2018 (Fig. 1). Fluazaindolizine at 1.12 kg/ha, 2.24 kg/ ha and fluazaindolizine + oxamyl significantly reduced nematode abundance by 73%, 62%, and 69%, respectively, compared to untreated control. Among treatments that included fluazaindolizine, there was no significant variation in level of suppressing SRKN abundances in 2018. None of the other nematicide treatments significantly reduced SRKN abundances relative to control in 2018.

**Figure 2:**
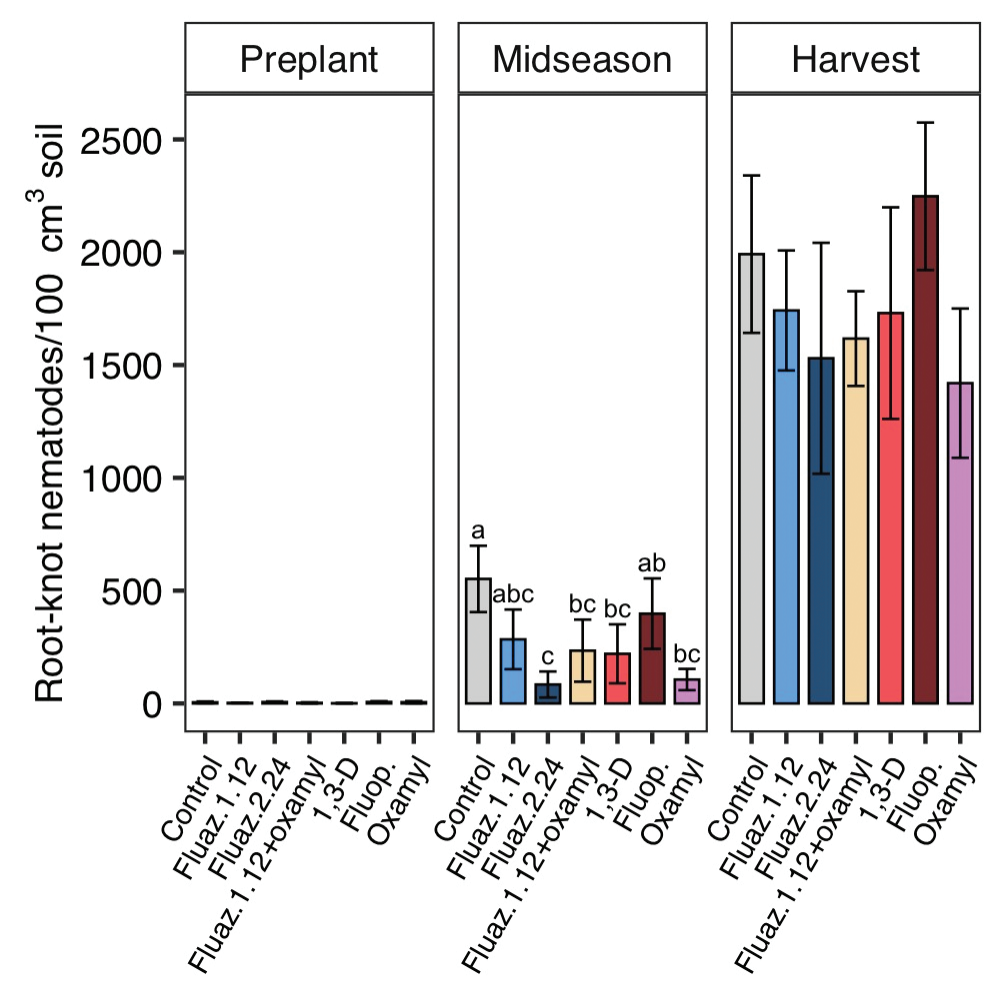
Nematicide effects on *Meloidogyne incognita* J2 soil abundances at preplant, midseason, and harvest in 2019 trials. Within each subfigure, means with different letters are significantly different (*P* < 0.05) based on Fisher’s protected LSD. “Fluaz. 1.12” and “Fluaz. 2.24” indicate fluazaindolizine at 1.12 kg a.i./ha and 2.24 kg a.i./ha, respectively. “Fluop.” Indicates fluopyram.

In 2019, the initial nematode pressure was low with four SRKN juveniles/100 cm^3^ soil, but increased rapidly during the growing season. At midseason in 2019, all nematicide treatments decreased SRKN soil abundances significantly compared with untreated control, except for fluazaindolizine at 1.12 kg/ha and fluopyram (Fig. 2). Fluazaindolizine at 2.24 kg/ha, fluazaindolizine + oxamyl, 1,3-D, and oxamyl reduced RKN by 85%, 58%, 60%, and 81%, respectively, compared to untreated control. There were no treatment differences on SRKN populations at harvest in 2019.

**Figure 3:**
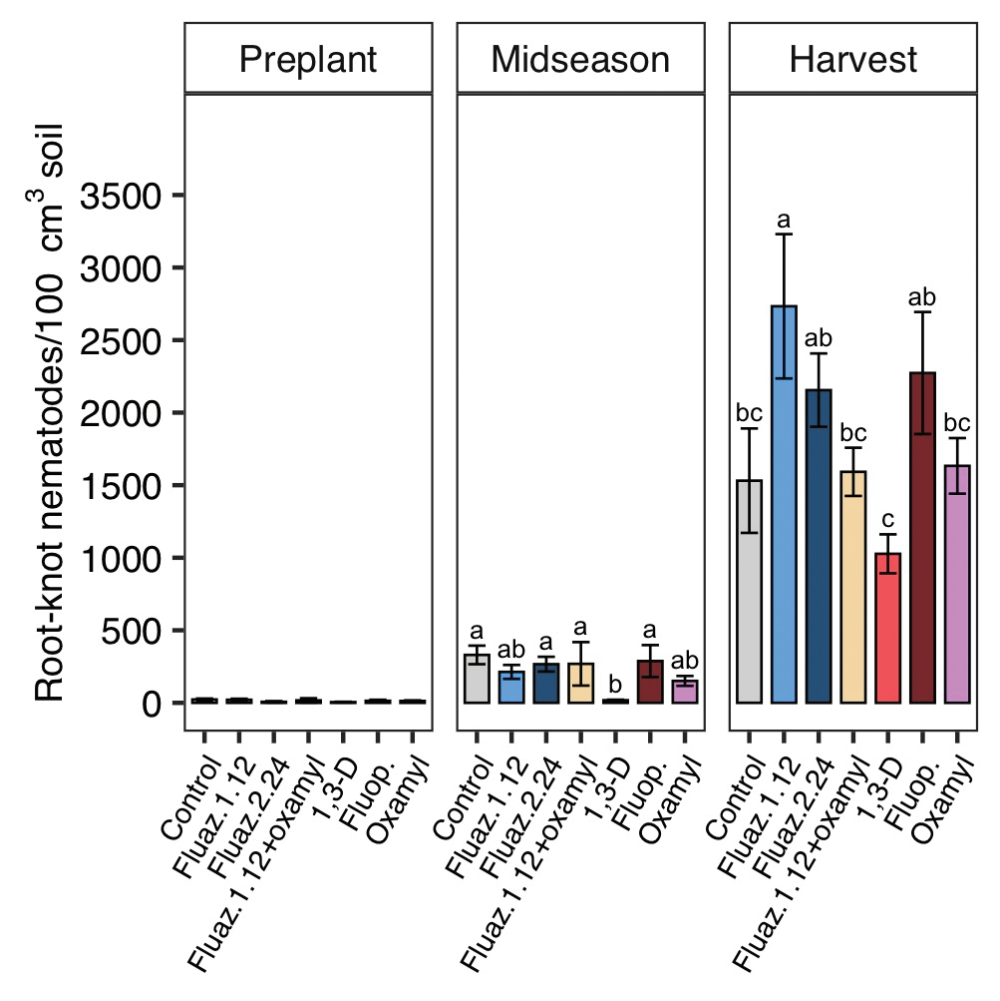
Nematicide effects on *Meloidogyne incognita* J2 soil abundances at preplant, midseason, and harvest in 2020 trials. Within each subfigure, means with different letters are significantly different (*P* < 0.05) based on Fisher’s protected LSD. “Fluaz. 1.12” and “Fluaz. 2.24” indicate fluazaindolizine at 1.12 kg a.i./ha and 2.24 kg a.i./ha, respectively. “Fluop.” Indicates fluopyram.

In 2020, the initial nematode pressure was low with 14 SRKN juveniles/100 cm^3^ soil. There was a substantial increase in RKN population throughout the 2020 growing season. At midseason in 2020, 1,3-D significantly decreased RKN population by 95% compared to untreated control (Fig. 3) and was the only treatment that affected SRKN population at that time. At harvest in 2020, none of the nematicide treatments significantly reduced SRKN soil abundances relative to untreated, but 1,3-D significantly reduced SRKN soil populations compared with fluazaindolizine at 1.12 kg/ha and 2.24 kg/ha and fluopyram.

### Nematicide non-target effects on free-living nematodes

**Figure 4:**
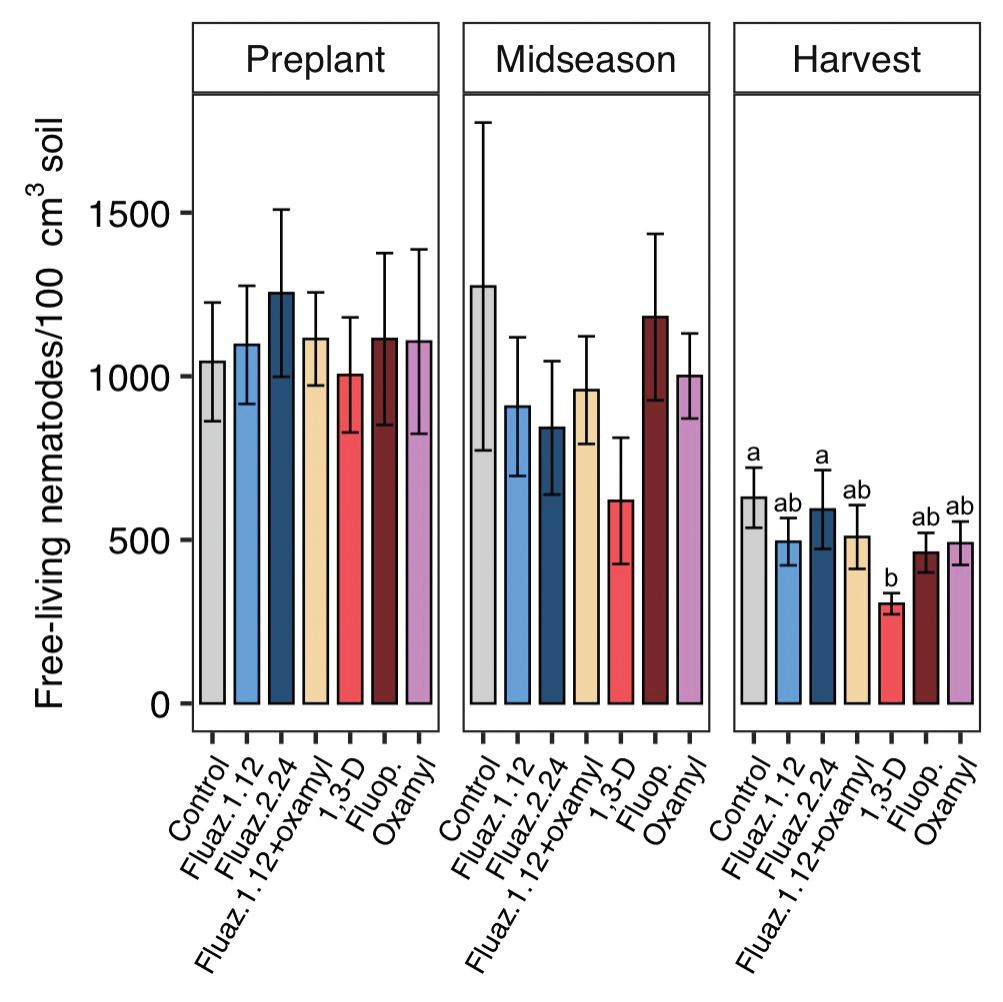
Nematicide effects on free-living nematode soil abundances at preplant, midseason, and harvest in 2018 trial. Within each subfigure, means with different letters are significantly different (*P* < 0.05) based on Fisher’s protected LSD. “Fluaz. 1.12” and “Fluaz. 2.24” indicate fluazaindolizine at 1.12 kg a.i./ha and 2.24 kg a.i./ha, respectively. “Fluop.” Indicates fluopyram.

**Figure 5:**
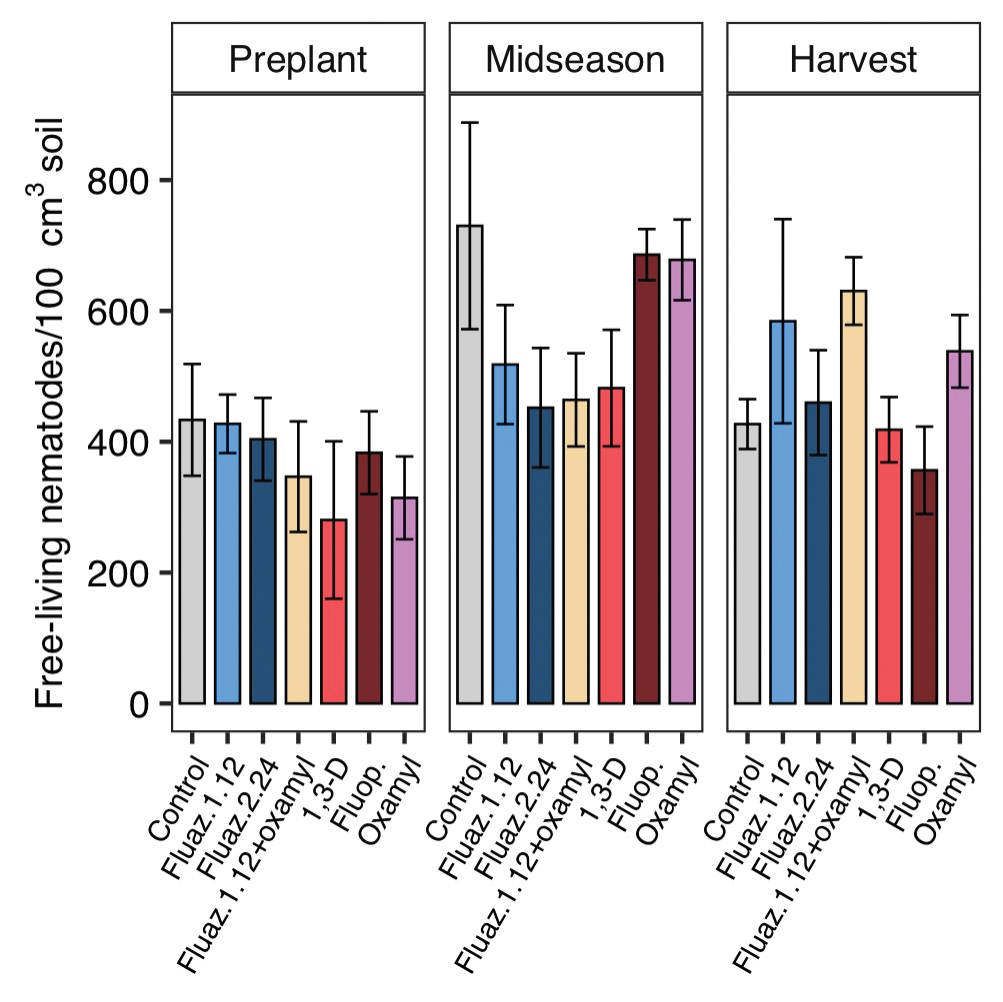
Nematicide effects on free-living nematode soil abundances at preplant, midseason, and harvest in 2019 trial. Within each subfigure, means with different letters are significantly different (*P* < 0.05) based on Fisher’s protected LSD. “Fluaz. 1.12” and “Fluaz. 2.24” indicate fluazaindolizine at 1.12 kg a.i./ha and 2.24 kg a.i./ha, respectively. “Fluop.” Indicates fluopyram.

**Figure 6:**
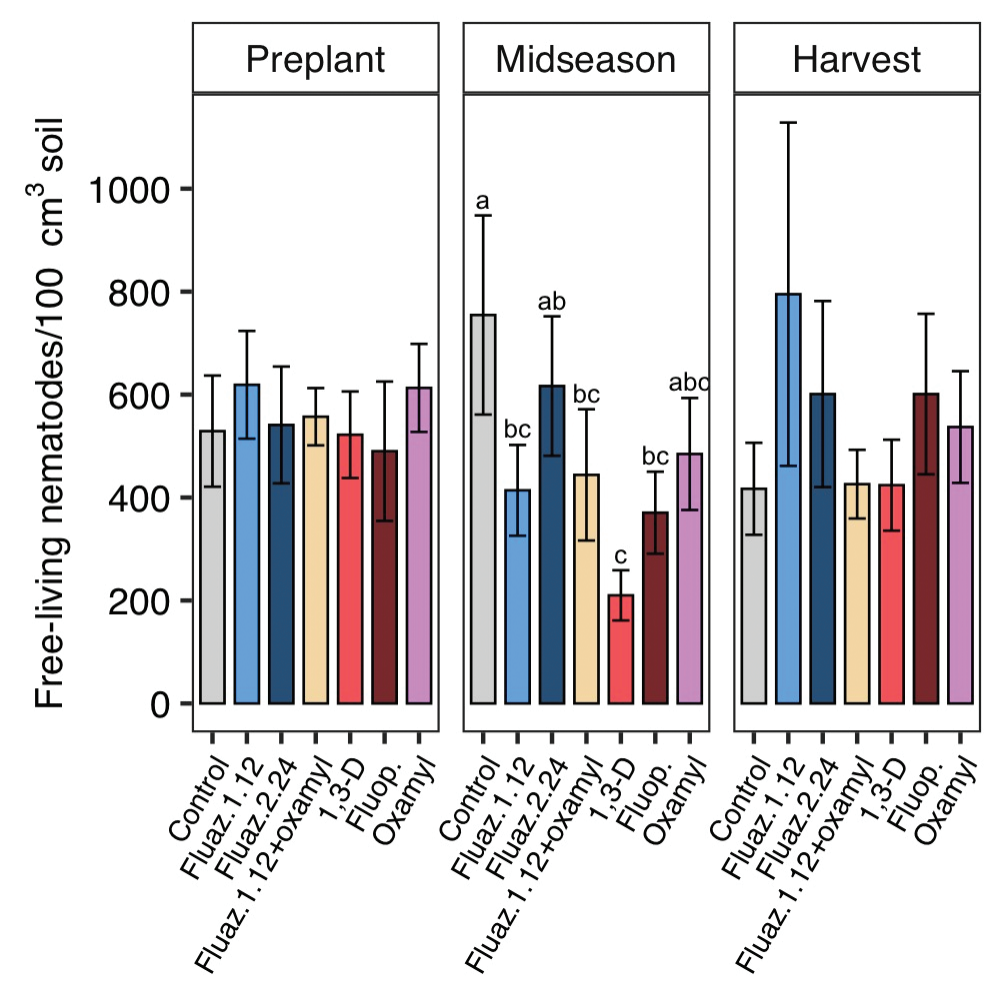
Nematicide effects on free-living nematode soil abundances at preplant, midseason, and harvest in 2020 trial. Within each subfigure, means with different letters are significantly different (*P* < 0.05) based on Fisher’s protected LSD. “Fluaz. 1.12” and “Fluaz. 2.24” indicate fluazaindolizine at 1.12 kg a.i./ha and 2.24 kg a.i./ha, respectively. “Fluop.” Indicates fluopyram.

In 2018, free-living nematode soil abundances did not significantly differ by treatments at midseason. At harvest, 1,3-D reduced free-living nematode abundances by 52% compared with untreated control, and for other nematicide treatments, abundances were not significantly different from untreated control (Fig. 4). Treatments showed no significant effects on free-living nematodes (Fig. 5) throughout the 2019 trial. In 2020, all treatments decreased free-living nematode population at midseason except for oxamylalone and fluazaindolizine alone at the higher rate (Fig. 6). No treatment effects were found at harvest in 2020 (Fig. 6).

### Nematicide effects on tuber yield and root gall rating

**Figure 7:**
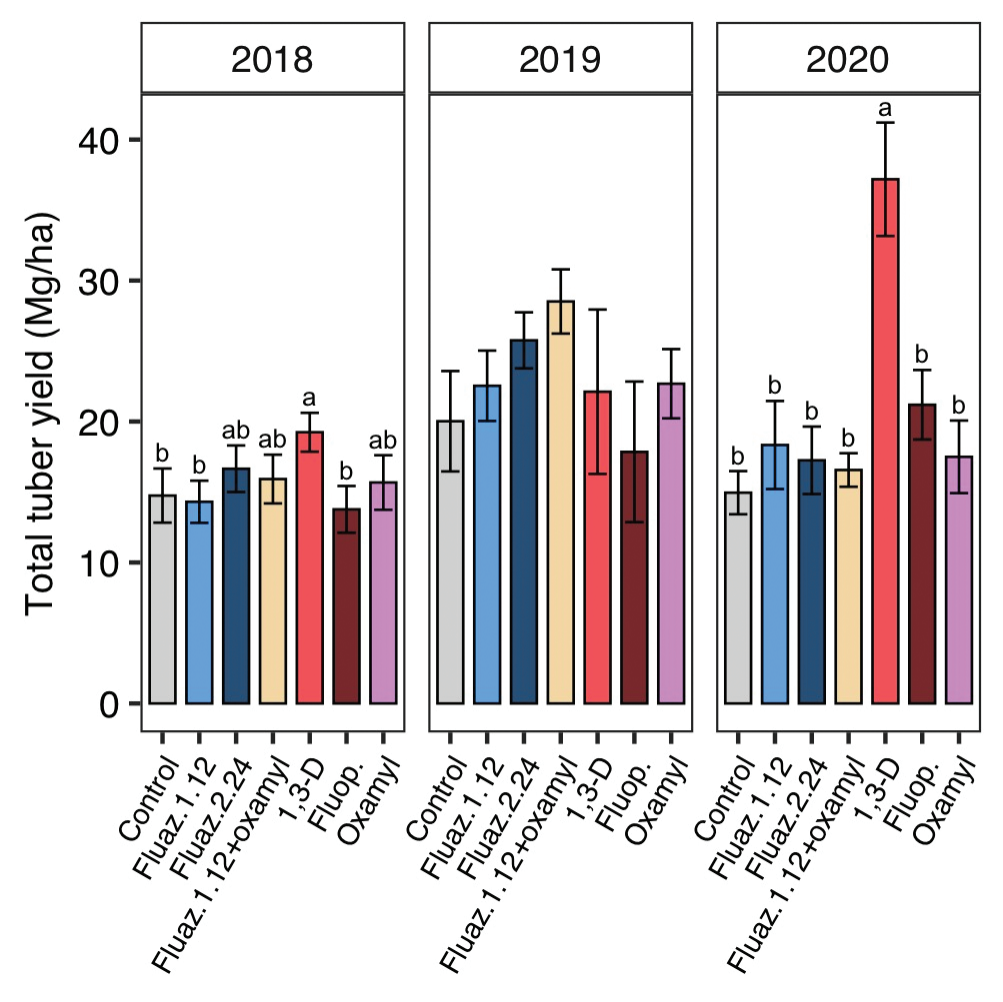
Nematicide effects on total sweet potato tuber yield in 2018, 2019, and 2020 trials. Within each subfigure, means with different letters are significantly different (*P* < 0.05) based on Fisher’s protected LSD. “Fluaz. 1.12” and “Fluaz. 2.24” indicate fluazaindolizine at 1.12 kg a.i./ha and 2.24 kg a.i./ha, respectively. “Fluop.” Indicates fluopyram.

**Figure 8:**
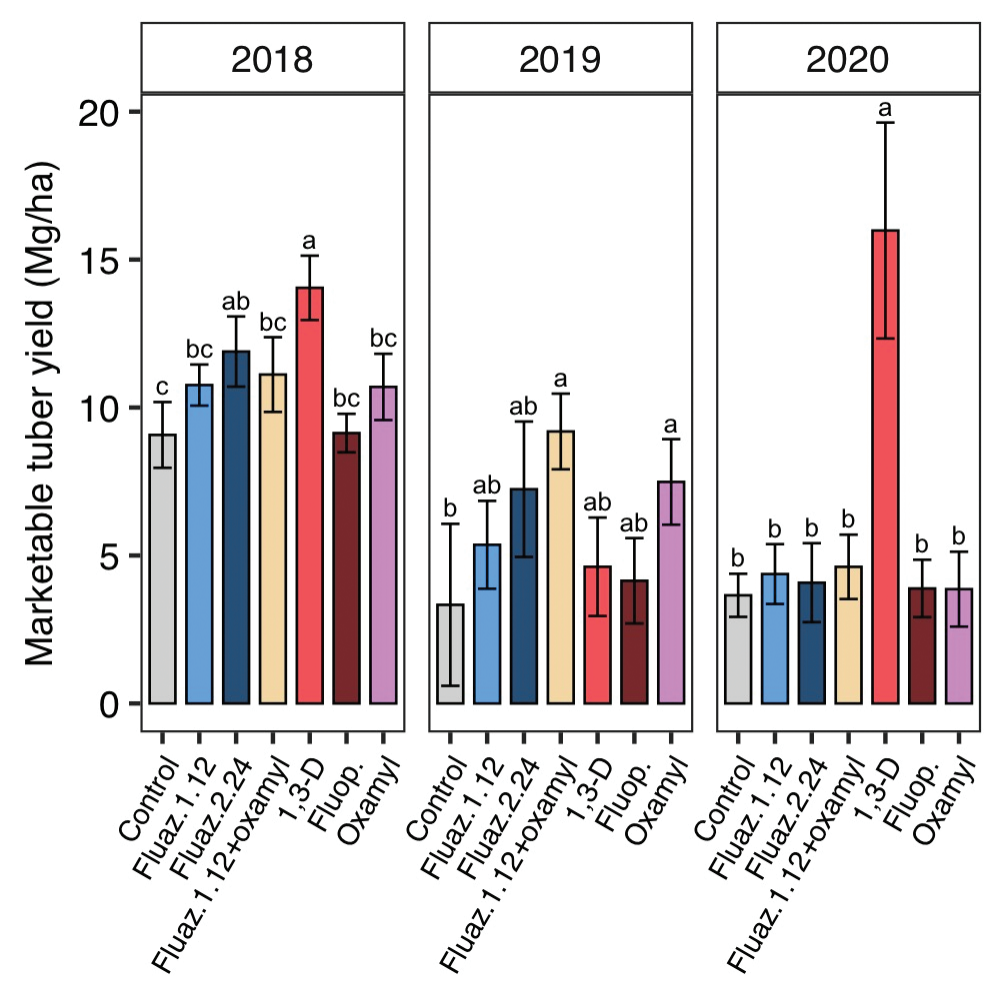
Nematicide effects on marketable sweet potato tuber yield in 2018, 2019, and 2020 trials. Within each subfigure, means with different letters are significantly different (*P* < 0.05) based on Fisher’s protected LSD. “Fluaz. 1.12” and “Fluaz. 2.24” indicate fluazaindolizine at 1.12 kg a.i./ha and 2.24 kg a.i./ha, respectively. “Fluop.” Indicates fluopyram.

In 2018, 1,3-D significantly increased total tuber yield compared with control, fluazaindolizine at 1.12 kg/ha, and fluopyram with a 30% yield increase compared to untreated control (Fig. 7). Treatment with 1,3-D as well as fluazaindolizine at 2.24 kg/ha increased marketable yield by 55% and 31%, respectively in 2018 (Fig. 8).

Treatments did not significantly affect total tuber yield in 2019 (Fig. 7), but significantly greater marketable tuber yield was observed with fluazaindolizine + oxamyl as well as oxamyl alone compared with untreated control (Fig. 8). Compared to untreated control, fluazaindolizine + oxamyl and oxamyl increased marketable yield by 177% and 126%, respectively. No significant treatment effects on tuber galling were observed in 2019 (Table 3). Among individual yield categories, only USDA 1 yield and shape outlier cull weight were significantly affected by treatments, but USDA 2, defects, and proportion defects were not (Table 3). Tuber yield of USDA 1 grade was significantly greater for fluazaindolizine + oxamyl than any other treatment, except it was not significantly different from fluazaindolizine at 1.12 kg/ ha. Similarly, shape outlier cull weight was greater for fluazaindolizine + oxamyl than untreated control or oxamyl alone with other treatments intermediate (Table 3).

**Table 3:** Sweet potato tuber yield (Mg/ha) by grade category and tuber gall rating as affected by nematicide treatments in 2019 and 2020.^a^

Treatment	USDA 1 (Mg/ha)^b^	USDA 2 (Mg/ha)	Defects (Mg/ha)	Size outliers (Mg/ha)	Percent defects^c^	Tuber galling (%)^d^
				2019		
Control	1.03 b	2.31	16.2	0.47 b	84	0.41
Fluazaindolizine	2.76 ab	2.60	16.29	0.87 ab	74	1.06
1.12 kg/ha						
Fluazaindolizine	2.40 b	4.84	17.56	0.94 ab	68	0.03
2.24 kg/ha						
Fluazaindolizine	4.46 a	4.70	16.82	2.49 a	60	0.21
1.12 kg/ha + oxamyl						
1,3-D	1.59 b	4.17	20.99	0.85 ab	76	0.67
Fluopyram	0.85 b	4.30	16.06	1.03 ab	73	1.14
Oxamyl	2.51 b	4.97	14.92	0.27 b	67	0.57
				2020		
Control	1.73 b	1.93 b	11.02	0.27	73 a	7.14 ab
Fluazaindolizine	1.57 b	2.80 b	13.71	0.25	75 a	8.51 a
1.12 kg/ha						
Fluazaindolizine	1.84 b	2.24 b	12.84	0.34	76 a	6.18 ab
2.24 kg/ha						
Fluazaindolizine	1.99 b	2.62 b	11.45	0.29	69 ab	8.22 a
1.12 kg/ha + oxamyl						
1,3-D	7.64 a	8.33 a	20.16	0.20	55 b	0.60 c
Fluopyram	1.84 b	2.04 b	17.03	0.27	81 a	3.93 bc
Oxamyl	1.99 b	1.86 b	13.35	0.27	78 a	6.50 ab

a Grade categories are based on USDA grade standards (USDA AMS, 2005) as summarized in the materials and methods. USDA 1 and USDA 2 are marketable grades. Defects are unmarketable due to poor quality tubers, primarily from wireworm damage. Size outliers are unmarketable, being outside USDA 2 requirements. ^b^Treatments with the same letters within the same column and year are not significantly different (Fisher’s protected LSD, a = 0.05). ^c^Percent culls (defects and size outliers) by weight relative to total yield. ^d^Percent tuber surface galled, average of 50 tubers assessed at harvest.

In 2020, 1,3-D significantly increased total tuber yield by 149% compared with untreated control (Fig. 7), while no other treatments performed differently from untreated control. There was a similar trend for total marketable tuber yield (Fig. 8), USDA 1 yield, and USDA 2 yield (Table 3). Although no significant treatment effects were found on defect and outlier cull weight, 1,3-D had a significantly lower proportion of defects compared with any treatment except fluazaindoline + oxamyl in 2020 (Table 3). Significantly lower tuber galling was observed with 1,3-D with 92% gall reduction compared to control (Table 3), while no other treatment reduced galling significantly.

## Discussion

Many field studies have demonstrated the efficacy of fluazaindolizine against RKN on a series of crops including carrots, cucumber, tomato, and squash (Becker *et al*., 2019; Desaeger and Watson, 2019; Hajihassani *et al*., 2019; Talavera *et al*., 2021; Zhang *et al*., 2021). However, to our knowledge, the efficacy of fluazaindolizine against RKN on sweet potato had not been previously investigated. This study showed that fluazaindolizine can be useful for managing SRKN soil populations, with efficacy in 2018 and 2019 but ineffective in 2020. A higher rate of fluazaindolizine – 2.24 kg a.i./ha – or combination of fluazaindolizine and oxamyl was somewhat more consistently effective than the low fluazaindolizine rate (1.12 kg/ha). The higher fluazaindolizine rate or fluazaindolizine-oxamyl mixture decreased soil SRKN in 2 of 3 yr (2018 and 2019), but the low fluazaindolizine rate decreased yield only in 2018. Inconsistent nematicide performance is common, especially among non-fumigant nematicides, and these inconsistencies could be attributed to various factors, like soil environment, precipitation, temperature, and cultivar (Oka *et al*., 2013; Desaeger and Watson, 2019).

Fumigation with 1,3-D was the most consistently effective nematicide at managing SRKN soil populations with efficacy in all 3 yr of testing. Past research also indicated that 1,3-D effectively managed RKN in sweet potato (Averre *et al*., 1975; Chalfant *et al*., 1992; Smith *et al*., 2009). Based on this study, oxamyl alone can also be effective at reducing SRKN soil abundances, but is also inconsistent, having efficacy in only 1 of 3 yr tested. While oxamyl is registered for RKN control on sweet potato and the effect of oxamyl on various RKN in various crops has been demonstrated (Wright and Rowland, 1982; Gugino *et al*., 2006; Peiris *et al*., 2021), efficacy of oxamyl against SRKN in sweet potato is minimal, so this study provides important efficacy data. Although many studies have proved the efficacy of fluopyram against RKN on various crops (Faske and Hurd, 2015; Jones *et al*., 2017; Dahlin *et al*., 2019; Hajihassani *et al*., 2019; Ji *et al*., 2019; Grabau *et al*., 2021), it showed little value for managing SRKN in this study. The application methods used in this trial could have contributed to the lack of fluopyram efficacy in this trial as it was only applied through chemigation in 1 of 3 yr of this study and that is the current label recommendation as well as the method used in most studies where fluopyram has been effective (Ji *et al*., 2019; Grabau *et al*., 2021). However, fluopyram was not effective in the 1 yr it was applied as a drench, suggesting that a change in application methods may not improve efficacy in this situation. Further testing would be needed for evaluation.

Aside from which chemistries are most effective, this study provided other insights into SRKN management in sweet potato. Study results indicated that even when a moderately resistant cultivar (“Covington”) is grown, applying nematicides could improve SRKN population control, particularly at the end of the season when SRKN populations were increased. Even with a resistant cultivar, it is still valuable for growers to include a nematicide in their control program. With susceptible cultivars (2019 and 2020), nematicide efficacy was most prominent at midseason, which is common. By harvest, SRKN soil populations were substantial and nematicides did not differ from control by the end of the growing season. This shows that with a susceptible cultivar, even if SRKN population was suppressed by nematicides at the beginning of the season, the protection could not last throughout the whole growing season, and SRKN reinfestation is occurring in the soil. While nematicides can still be effective at protecting sweet potato yield, they would not be effective for managing SRKN for a subsequent crop, meaning that nematicide application must be done each growing season unless combined with other management practices.

Nematicides also varied in their efficacy at increasing sweet potato yield. Fumigation with 1,3-D was the most effective nematicide, increasing total and marketable yield relative to untreated in 2 of 3 yr (2018 and 2020). No other nematicide significantly increased tuber yield, although oxamyl alone, fluazaindolizine at 2.24 kg/ha, and fluazaindolizine + oxamyl each increased marketable yield in 1 of 3 yr. Neither the low fluazindolizine rate (1.12 kg/ha) nor fluopyram ever significantly increased sweet potato yield. Similar to SRKN soil population results, 1,3-D was the most consistently effective nematicide for increasing yield among those tested, but fluazaindolizine (at 2.24 kg/ha), oxamyl, or combinations of the two can also be effective, albeit less consistently. In general, yield benefits of nematicide application roughly corresponded to SRKN population control, in that for a given year, the nematicides that best managed SRKN soil abundances also yielded the best. This suggests that most of the yield benefits of nematicide application were due to SRKN management.

Other factors, namely wireworms (*Conoderus* spp.), serious pests in north Florida sweet potato production (Seal *et al*., 2020), which were present in this study, could have also contributed to yield response. Wireworms feed on sweet potato tubers causing scars and holes, primarily affecting tuber quality (Seal *et al*., 2020), although it is suggested they reduce bulk tuber quality under high pressure (Chalfant *et al*., 1992). Both 1,3-D and oxamyl are reported to have activity against wireworms (Chalfant *et al*., 1992; Arrington *et al*., 2016), but neither fluopyram nor fluazaindolizine are known to have any insecticidal activity. From results of this trial, there was weak evidence that 1,3-D had better wireworm control than other products in 2020, based on a decreased proportion of defects, which were primarily from wireworms. None of the other products affected defects.

All nematicides tested had non-target effects on free-living nematodes, although not in every sampling date or year. Fumigation with 1,3-D had the most consistent negative impacts on free-living nematodes (2 of 3 yr), which agrees with many previous studies (Collins *et al*., 2006; Grabau *et al*., 2020). Fluazaindolizine, oxamyl, and fluopyram all negatively impacted free-living nematodes, but only in 1 of 3 yr of the study (2020). A lab study showed that sensitivity to fluazaindolizine differed by free-living nematode species and the fitness of some species of bacteria-feeding species were negatively impacted (Talavera *et al*., 2021), but in general, free-living nematode fitness were not adversely impacted (Thoden and Wiles, 2019; Talavera *et al*., 2021). Our results are generally in line with these findings, and greater sensitivity of free-living nematodes to fluazaindolizine in 2020 could be a result of a greater proportion of bacterivores at the site in 2020, since this trophic group is more sensitive to fluazaindolizine (Talavera *et al*., 2021). One limitation of this study was that we evaluated only nematicide impacts on total free-living nematode abundances, so nematicide impacts on individual nematode trophic groups or genera were not quantified. For a full assessment of non-target effects of fluazindolizine and other new nematicides on the free-living nematode, field evaluation at a finer ecological and taxonomic resolution is needed.

Various results have been reported on effects of fluopyram on free-living nematodes. Grabau *et al*. (2020) reported no effect of fluopyram on free-living nematodes in peanut production, while Waisen *et al*. (2021) and Waldo *et al*. (2019) reported negative effect of fluopyram on free-living nematodes on tomato (*Solanum lycopericum*) and bermudagrass (*Cynodon dactylon*), respectively. Despite being an established chemistry, there is relatively little prior information on non-target effects of oxamyl. In tomato field production, oxamyl always had non-target effects on free-living nematodes (total soil abundance) when it effectively managed SRKN (Grabau *et al*., 2021). Similarly, in microcosm (Carrascosa *et al*., 2015) and field studies (Ntalli *et al*., 2018) in tomato production, oxamyl also negatively affected free-living nematodes. An ideal nematicide would be specific to target plant-parasitic nematodes, without adverse effects on free-living nematodes. In this study, none of the nematicides tested met that criteria, with all nematicides having some non-target effects and the most consistently effective nematicide against the target SRKN also having the most consistently negative impacts on free-living nematodes.

In summary, 1,3-D was the most consistent nematicide for SRKN management in sweet potato. Fluazaindolizine – particularly at a higher rate of 2.24 kg/ha – and oxamyl can be effective non-fumigant nematicides for SRKN sweet potato production, but are not as consistent as 1,3-D fumigation. Fluopyram did not show much efficacy, but more testing with drench application methods is needed. With the increasing concerns for environment protection, searching for new nematicides that can cause less harm to soil, water, and microbes are in urgent need. Our study showed that fluazaindolizine can be effective for reducing SRKN and can serve as a potential alternative to soil fumigation for sweet potato growers.
